# Syphilis and Marriage

**Published:** 1893-02

**Authors:** I. Neumann, G. A. Himmelsbach

**Affiliations:** Buffalo, N. Y.; Late Resident Physician to the Buffalo General Hospital; 137 W. Tupper Street


### Translation.

### SYPHILIS AND MARRIAGE.

By PROF. I. NEUMANN, Translated from *Wiener Medizinische Wochenschrift*, No. 23, June 4, 1892.

By G. A. HIMMELSBACH, M. D., Buffalo, N. Y.

Late Resident Physician to the Buffalo General Hospital.

### SHOULD A SYPHILITIC PERSON BE ALLOWED TO MARRY ? AND UNDER WHAT CIRCUMSTANCES IS MARRIAGE ADMISSIBLE ?

I shall enter upon the discussion of a question which, according to Fournier, is, to a prominent degree, quite perplexing. It is a subject which embraces a number of the most diverse, difficult, delicate, and dangerous problems by which family affairs are often surrounded, a question which demands the greatest responsibility of a physician,—in fact, there is hardly a theme, or subject within the whole domain of medicine of such great importance to the individual, as well as the family, or even the state, as the one just mentioned to be treated upon.

Syphilis does not alone endanger the health of a married person ; it is even more dangerous to the vital conditions of the offspring, and it is the latter direction in which it especially develops its destructive and serious consequences. A very large proportion of abortions have their origin in the syphilitic state of a person. A great many children born of syphilitic parents bring syphilis into the world with them, or else they succumb to it a short time after birth, while, indeed, others may remain free from the appearances of the disease, and yet showing weakness of vitality, general degeneration, and will be carried off in a short time.

Upon the basis of observation, Fournier reports having seen 500 married people in a syphilitic state, by which there were 1,129 births presenting manifestations of the disease, and the rate of mortality of the offspring of these syphilitic parents was forty-two per cent. (*i. e.*, of all births), but according to further observation by the same author, among the poor patients who were delivered in the hospital St. Louis, (France,) this rate of mortality increased to eighty-four per cent., and according to Le Pileur, it reached the awful number of ninety-four per cent.—yes, even ninety-six per cent.—which disease, with a still increasing number of cases, was almost productive of a social panic. There is no wonder that the question has long since aroused the attention of the medical world, without arriving, up to date, to definite results. The great number of medical men who busy themselves with this subject, arrive at the most diverse and partly direct contradictory results and views. While Fournier, for instance, in reference to the matter of allowing matrimony, stands alone, with his pessimistic views, and demands, at least a space of three or four years before matrimony, and, furthermore, he demands from one-half to one year's time in which there is no appearance of the disease, and even then only allows matrimony when the disease was a mild attack, and which gave way easily to medical treatment, and, finally, if the respective individual has for three years gone through a thorough, systematic, intermittant treatment of six months each, with mercury and iodides.

Hutchinson, in expressing himself in reference to this subject, is much more sanguine. He allows a patient to marry after two years, and further states that he never has found any reason, in spite of numerous observations, to repent for this concession,—as mothers and children had always afterward been free from the disease.

Boeck even allows a patient to marry, although he had presented manifest symptoms of syphilis only a few months previous, and says he has never yet received reproach from any such cases.

Wolff and Cullerier finally deny altogether the infection of syphilis from the father, and contend that only when the mother suffers from the disease can the offspring (children) inherit it.

Now, then, the question: What standpoint shall we take in the midst of so varied and manifold views? Before I enter upon the response to this question, I shall, in the first place, introduce a number of cases as illustrations, and which will present questions for further discussion and explanation later.

CASE I. A youthful, strong individual had initial lesion three years ago, followed by eruption, and which disappeared after thirty applications of Ung. Hg. Since then no repetition and no sign of syphilis has appeared.

Case II. A man of middle age, lives in good social circumstances. The initial lesion and exanthem appeared two years ago ; then used mercurial inunction and disappeared. Six months later the exanthem reappeared, and a second use of the inunction caused it to disappear. Has been well since then, except has suffered from psoriasis of the ear, which still exists. Besides this psoriasis, and a moderate swelling of the lymphatic glands, there is no sign of the disease.

Case III. A man forty years old, extravagant liver, heavy smoker, and toper, and inclined to sexual excesses. Initial infection four years ago ; mercury treatment ; cure. In the course of the first two years he had repeated outbreaks of the eruption, but, finally, all disappeared with mercury treatment. Since then, free from repetition, free from psoriasis of ears, hands or feet, but swelling of lymphatic glands on both sides.

Case IV. A female patient, twenty years, belonging to the poor class ; initial infection and exanthem three years ago ; mercury treatment ; cure. Since that time several small nodules have appeared on the genitals. Mercury treatment given in some form or other. The past year no repetition, no return, and, at present, no sign of syphilis, except multiple swelling of glands in groins.

These different cases in their reference to the individual suffice to show that it is difficult, yes, is even impossible to lay down general methods of treatment, by which we may be directed under all circumstances and in all cases. The disease may appear in such a variety of forms. It is more natural, then, that in general the medicinal treatment must be chosen essentially according to the individual, and not to follow one definite scheme or plan, but to treat according to the nature of each individual case.

It is also understood, and has been proven daily, by experience, that syphilis of one (husband or wife) in matrimony reflecting or producing its effects upon the other as well as upon the offspring, must be of different form, and varies according to the sex, age, constitution of the diseased person, and, also, with respect to the form, appearance, and age of the respective infection.

In the first place, let us make a difference between syphilis in man and syphilis in woman. It has, indeed, been shown by experience, and to great satisfaction, that, on account of the greater number of cases in which man brings syphilis into matrimony, (according to Fournier, 487 out of 500 cases,) syphilis of the father, it is, which effects the most serious results upon the wife and the offspring; this statement, however, only refers to the social relation, and not to the seriousness or gravity of the disease of the individual. On the contrary, syphilis of the mother, though somewhat less in the better society, shows itself by its effects upon its offspring by far more serious and dangerous perpetuation. The reasons are well defined. Syphilis in man is communicable in a two-fold direction:

1. By infecting the wife, and by her communicating it to the child.

2. By infecting the child alone, in some way, without affecting the mother, who later may exceptionally be infected by the child.

In reference to the first group of cases mentioned, the conditions of development of the disease in the child are the same as they are in the mother, who is directly infected. Inasmuch as the infection of a wife, through a syphilitic diseased husband, results only in a part of all the cases, the proportion of percentage of the offspring who are thus infected is much more favorable than with the mother who is directly infected by the disease.

The second class of cases comprises those in which the child is infected with the disease through the father, while the mother seems to remain healthy (intact, so to speak). To these observations belong those cases in which the father presents at a glance a variety of positive advanced symptoms; consequently, the disease is contracted through sexual intercourse, as the sperm of the father contains the syphilitic poison. In this case the prognosis is more favorable to the child than when the mother suffers from tertiary, or constitutional, syphilis. The harmful influence and its consequences are effected, therefore, only at the act of coition. From that very moment no more syphilitic matter enters the fetus by the placental circulation; it, therefore, presents a by far more favorable chance than the fetus of a syphilitic diseased mother, the development of which, in case of the latter, is to the highest degree impeded by the constant supply of syphilitic infected blood; that the blood of the mother has an influence of this kind upon the child, is purely shown by the syphilitic state in which we find children that have been exclusively infected by the blood of the mother. I shall present reasons further along, to show that syphilis in women is more dangerous than that of man. A few statistical points may find place here; they quite agree with the aforesaid statements.

Fournier collected statistics of 500 births where the father was diseased ; thirty-seven per cent. of which inherited the disease, and twenty-eight per cent. died ; and where the mother was diseased, eighty-four per cent. inherited, and sixty per cent. died ; and where both parents were diseased, ninety-two per cent. inherited, sixty-eight per cent. died. This shows that the contagiousness and the mortality were more than two-fold when the mother was infected than when the father. The following figures, which are the result of observation for several years at my own clinic, also show the disastrous influence on the part of the mother upon her offspring. Of 123 mothers treated in my clinic, and who were diseased before conception, 208 children were born ; of these only sixty-one escaped the disease, (*i. e.*, were healthy,) while 147, or over seventy per cent., of the children inherited and suffered from the disease. Seventy-one of those mothers were in the first or primary stage, and these gave birth to ninety-six diseased children ; of these ninety-six sick children, only twenty remained healthy afterward, or about twenty per cent. Fifty-two mothers who were in the third tertiary stage, gave birth to 109 diseased children, of which forty-one remained healthy, or little over thirty-seven per cent.

The last mentioned figures lead us now to a certain point which is of great importance as regards the influence of syphilis of the father and of the mother. I mean by that, the degree of infection of the child varies according to the form or stage of the disease found in either or both sexes. As it is well known, there are in most cases an early so-called secondary stage in which the most important sign is the moist chancre, which for infection of the disease from one person to another is the most dangerous. The infection in most cases takes place at coition, from a fresh or late efflorescence, or even by a kiss through fresh erosions of the mucous membrane of the lips.

The products of a syphilitic ulcer are equally virulent in both sexes—with women, especially, at the sexual part, and with men, the mucous membrane of the mouth. To these individuals we have to direct our special attention, and, particularly, to those who wish to marry within that dangerous period, it is necessary to promptly interdict, not only on account of the danger of infection of one person to another, but still more on account of the exceeding great danger of transmitting the disease to the offspring.

As these efflorescence make their appearance in the early part of the disease, it is evident that especially the first three years of the disease must be considered the most dangerous, and in which marriage should not be allowed, and of these three years, it is the first year, according to Fournier, in which the mortality reaches one-half the whole number of cases, while, according to the same author, the mortality of the second year, amounts only one-third of the first; that of the third year one-half of the second; and that of the fourth year is less than half of that of the third; while the degree of virulence of the second stage of both sexes seems to remain very nearly the same as regards their results. The results of a later so-called third stage of the disease are quite different, as regards the degree of virulence, upon man and woman, as we have before alluded to in the above mentioned statistics, which show that children born, in which the mother was in the third stage of syphilis, the rate of over sixty-three per cent. inherited the disease. The circumstances, when the woman is the one diseased, are by far more unfavorable than when the man is diseased, not but what the danger of infection by man is at least just as great,—yes, probably far greater than with the woman,—but in case of the latter the growing fetus is essentially in more unfavorable surroundings or circumstances. In the woman, we have also a third stage, which is much more dangerous to the offspring than the third stage of man, and so we see the results of marriages in which the woman alone is specifically infected, that the most part of the fetuses die in utero, or are delivered with extravagant marks of the disease, or if they are born without such marks, in spite of all that can be done, will die in a short time of the disease or a general grave dyscrasia. Therefore, matrimony can be sooner allowed to man who has been infected by the disease than woman, because the forms or stages of the disease in the latter causes a hindrance to matrimony, while with the former it does not necessarily interfere; with a woman, it will be necessary that the infection must have long since passed away before matrimony can be allowed to her, while this is not the case with the man.

The moist chancre is found more frequently and is more permanent upon the sexual parts and organs in woman than with man, with whom we have already seen the sexual parts are not so often the seat of infection as the mucous membrane of the lips and mouth, hence, the former are a more important source of hindrance to matrimony.

It is, of itself, understood that the longer the lapse of time from the moment of infection, the more favorable are the circumstances, and to draw a fine line of mathematical exactity, no one is able to do. It is so indefinite, it fluctuates, or varies according to the character of the disease and the constitution of the individual, so that it will not be out of place to allow matrimony under some circumstances after an elapse of one and one-half to two years, while with others it cannot be done with safety short of ten years. Nevertheless, it is of importance, and to the highest degree of great responsibility on the part of the physician, to consent to the points which Fournier has laid down. I should like to suggest, for the great majority of cases, a space of three years from the time of primary infection, and at least one full year free from all appearances of the disease. When this condition of affairs is found to exist, without any symptom of the disease to be seen, and from the beginning a faithful and long treatment was pursued, matrimony may be allowed to a man, not, however, without making known to the candidate the possible consequences of his matrimony. One should also be very cautious to state a favorable result with surety, and to take upon himself the responsibility of all the consequences of such a step. Besides this, the respective individual should be advised, before entering into matrimony, even when apparently free from syphilitic appearances, to undergo a systematic anti-syphilitic treatment, and, indeed, if possible, the inunction treatment. This measure with men who are not candidates for marriage, at a time when no symptoms are present, is unnecessary. Still, it appears necessary when the matrimonial step is taken to continue this treatment. I speak of such cases which are cited as manifold in medical literature, in which, after matrimony, those men who had been syphilitic for a length of time, still at the time of matrimony presented no sign of the disease; year after year one abortion followed the other, and no children of health and vitality were born until the respective husband had undergone a course of treatment. In general, the question as to whether the respective person had been rationally treated when in the syphilitic state, is one of the most important in medical practice, as it has been statistically shown and daily approved by testimonials, that after a thorough treatment, the dangerous results of the disease appear by far more seldom and with less intensity than when those who had either not been treated at all or, if so, very care lessly.

When Fournier, however, goes a step further and demands, as a pre-requisite to matrimony, that the respective patient has to undergo, for several years previous, a prolonged intermittent treatment, I claim this complicating treatment is superfluous and molesting to the individual, and is injurious to the same, not only physically, but even mentally. In order to attain a proper idea as to whether the respective individual should marry or not, I think it is first and most important to state whether and in which manner the same is infected by the disease at the time. This statement Fournier thinks is quite impossible. How is it, then, when the disease has been energetically fought against for a long time, no trace or existence of the disease can be found anymore? That such cases happen where syphilis has been caused to disappear entirely, is shown by foreign observations as well as our own.

I am, at this moment, in the position to name more than sixty cases, in which, after only one course of treatment, partly in my own clinic and partly in private practice, who, after a space of from one to sixteen years, introduced themselves at my clinic again without any sign of syphilis. About a certain number of cases I am unable to furnish the necessary figures. A by far greater number of cases may have disappeared from my memory, from the fact that I never saw the patients again.

137 W. Tupper Street.

